# The Marking of the Domestic Animals, and the Use of the Patent Marking-tongs

**Published:** 1897-06

**Authors:** Branchli Vigoltengen


### ABSTRACTS FROM FOREIGN JOURNALS.

### GERMAN.

UNDER THE DIRECTION OF J. PRESTON HOSKINS,
PRINCETON, N. J.

THE MARKING OF THE DOMESTIC ANIMALS, AND THE USE OF THE PATENT MARKING-TONGS. (By Branchli Vigoltengen.) As is well known, different tribes of Indians and South Sea Islanders adorn their bodies with tattooing-marks. Besides forming an adornment, many of these designs serve as the sign of race, family, or rank. Sometimes they are employed as a reminder of treaties that have been made or of particular events. Even to-day sailors and soldiers have themselves tattooed, mostly upon the arms, and this practice, according to the newspapers, has come into fashion lately among English lords.

Our domestic animals, especially the horse, were also marked in ancient times, either with a view to being able to recognize the animal again or to designate its breeding and family. This marking was largely practised by the Greeks and Romans especially to distinguish animals of different lineage, some of which had attained unusual celebrity.

Alexander the Great's celebrated horse, Bucephalus, is said to have received his name from an ox's head, which was branded upon his shoulder, as well as upon all other horses which were descended from a famous Thessalian stud.

At present the marking of horses on stud-farms and in the army is a pretty general practice, usually as a means of establishing the pedigree and facilitating registration.

Marking of animals, however, is useful not only for these purposes, but it plays a very important part also in the control of epidemics and boundaries.

According to the object and kind of animals the marking is performed in different ways. As a transitory mark, often the hair on some particular spot is cut off in the shape of a letter or figure, or less often burned off, or a mark is made with colored chalk, as is the case in many of our cattle-markets. But to make a lifemark, or at least one which will endure for a long period, the following different methods have been used:

1. Cutting out of pieces of skin.

2. Making incisions (nicks) in the ears and piercing the same.
3. Tattooing.
4. Branding and applying caustics.
5. Application of marks.

The first method—cutting out pieces of skin—is much practised on young animals in Hungary; a permanent scar remains at the place where the cut is made.

The second method—making cuts in the ears—is performed in many places and in many different ways. In Spain, mares which are no longer useful for breeding purposes are marked by cutting off a piece of the right ear. In France, horses which had been mustered out of army service used to be marked by splitting one of the ears so that they would be easily recognized by the experts who had to examine the new supplies. This practise was abolished, however, as the value of the animal was thereby deteriorated.

In Switzerland, those horses which have been rejected as unfit for cavalry service receive a nick on the outer edge of the left ear; the operation is performed with tongs.

Cattle, especially in the Alps, and sheep are often marked by making a nick or incision in the ear. Besides the nick on the edge, perforations in the ear itself are often made in sheep, and the mark has an especial meaning, according to the number and position of the nicks and holes. Boring the ear is much used in the case of swine.

Tattooing in cattle and sheep is performed on the internal hairless surface of the ears; in swine on the external surface of the same. By means of the so-called tattoo-tongs, which consist of numbers, letters, etc., composed of sharply-pointed needles, a mark is punched into the skin of the ear, and later coloring-matter is rubbed into the spot. Although this method has its imperfections, it is much used upon white swine.

The hot iron is used very often to make both a transitory and a permanent mark. In the first case, the mark is generally burned upon the hoof or horns; in the second, on the skin. The iron must be red-hot and applied quickly with light pressure. The foals in our country which are sired by the stallions belonging to the Swiss Confederation have the national cross burned upon the left rump.

The application of the hot iron brings several inconveniences in its wake. On the hoof of horses the marks descend with the growth of the hoof, and are finally cut away by the blacksmith. On the horns the marks are frequently rubbed off against various objects, so that in both cases it is necessary to renew them every few months. On the other hand, these marks not seldom become obliterated and illegible, particularly if the sign is a complicated one, consisting of narrow and intricate lines, for that reason the marking should be as simple as possible.

A further disadvantage of branding on the skin arises when the iron is too hot and is held on too long with strong pressure; the places branded run together, suppuration sets in, and makes an illegible and disfiguring scar. If the iron is not hot enough or the pressure too weak and irregular, the brand is not sufficient, and after a short time will be obliterated by the growing hair; a second branding on the same spot is not practicable, as the operator can seldom strike the same spot exactly. Burning, too, is a painful operation, and on sensitive animals not always to be carried out without danger.

Instead of a hot iron, caustics or corrosive materials have often been used, especially the so-called Vienna paste, which consists of equal parts of calcium and potassium hydrates. This was usually mixed with water, so as to form a thick paste, and then applied to the wooden model of the mark to be made, or the model was dipped into the paste and then pressed against the spot where the mark was to be made, the hair having been previously removed. The paste is best prepared by dissolving the calcium hydrate in three to five parts of alcohol and then adding the corresponding quantity of potassium hydrate. When the paste is applied it can be moistened with alcohol in order to dry it more quickly. It can also be applied with a brush. This method was formerly common in France, and is clean, regular, and less painful and dangerous than the hot iron; on the other hand, it is more troublesome, and sometimes a second application must be made.

Having named the different methods of marking and their advantage and disadvantage, we now turn to another method of marking cattle, namely, that of placing some sign upon the ears.

Branchli's first attempts were made with Alpine cattle. He used round pieces of tin stamped with numbers, which were fastened on by wire piercing the ear and clasped on the other side. In most cases the marks were lost while the cows were up in the Alps, the wire breaking by being rubbed against various objects.

Accidentally the "Rivet tubulaire double" (double tubulur rivet) came to his notice, which he at once decided to try as a means of fastening the marks. First of all a hole corresponding to the rivet must be made in the ear, and afterward the double rivet must be pressed together by means of a pair of tongs, so as to fasten the two disks on the ear. For this purpose he had a pair of tongs made which could first be used to make the hole and then to press the rivet together. He then experimented on different cattle. The result was various. In some cases the marks held, in others they fell out. There were two reasons for this: either the double rivet had not been pressed together tightly enough, and so parted of itself, or it had been pressed together too tightly. In the latter case it caused a severe inflammation, with suppuration and necrosis round about the hole, and the latter became so large that the back plate of the rivet slipped through.

To mark successfully the hole should be made with the tongs about 5 to 7 cm. from the apex of the ear and 4 to 5 cm. from the under edge, where the minimum circulation of blood is found. The puncture should be painted with tincture of iodine, creolin, or carbolic water, and the rivet put in some days afterward. The two plates of the rivet should not be more nor less than 1 cm. from each other. In the first case the rivet may come out; in the latter the ear is compressed too much. For the first week, or until the ear heals, it is to be bathed with creolin or carbolic water, and the marks should be carefully revolved. The marks he has had made of aluminium 1 mm. in thickness, and found it very satisfactory. —*Schwaizer-Archiv fur Tierheilkunde,* January and February, 1896.

